# Polymorphisms in *FADS1* and *FADS2* alter plasma fatty acids and desaturase levels in type 2 diabetic patients with coronary artery disease

**DOI:** 10.1186/s12967-016-0834-8

**Published:** 2016-03-22

**Authors:** Si-Wei Li, Jin Wang, Ying Yang, Zhi-Jie Liu, Lin Cheng, Huan-Yu Liu, Pei Ma, Wan Luo, Song-Mei Liu

**Affiliations:** Center for Gene Diagnosis, Zhongnan Hospital of Wuhan University, Donghu Road 169#, Wuhan, 430071 People’s Republic of China; Scientific Research Center, Shanghai Public Health Clinical Center, 2901 Caolang Road, Jinshan District, Shanghai, 201508 People’s Republic of China; Key Laboratory of Combinatorial Biosynthesis and Drug Discovery (Wuhan University), Ministry of Education, Wuhan University School of Pharmaceutical Sciences, Wuhan, 430072 People’s Republic of China; Department of Clinical Medicine, Hubei University of Medicine, Hubei, 442000 China

**Keywords:** Coronary artery disease (CAD), Type 2 diabetes (T2D), Fatty acids, Desaturase, Polymorphism(s)

## Abstract

**Background:**

To explore whether plasma fatty acids and SNPs in the fatty acid desaturase (*FADS*) gene associated with type 2 diabetes (T2D) and coronary artery disease (CAD).

**Methods:**

In this cross-sectional study, we utilized gas chromatography–mass spectrometric analysis and the high-resolution melting method to detect plasma fatty acids and SNPs respectively (rs174537G>T, rs174616C>T, rs174460T>C, and rs174450A>C) in 234 T2D, 200 CAD, 185 T2D&CAD patients, and 253 healthy controls.

**Results:**

We found that T2D&CAD patients had the highest plasma arachidonic acid, dihomo-gamma-linolenic acid and delta-6 desaturase, and the lowest stearic acid, linolenic acid, and saturated fatty acids; plasma eicosapentaenoic acid and docosahexaenoic acid elevated in T2D patients, but significantly reduced in CAD patients. Moreover, T2D patients with rs174537 GG genotype were at risk of developing T2D&CAD (odds ratio (OR) 1.763; 95 % CI 1.143–2.718; *p* = 0.010), with elevated plasma LDL-cholesterol, arachidonic acid, and delta-6 desaturase.

**Conclusions:**

Our results show that SNPs in *FADS* gene (particularly rs174537) associate with plasma fatty acids and desaturase levels in patients with both T2D and CAD, which maybe increases the risk of CAD in diabetic patients.

**Electronic supplementary material:**

The online version of this article (doi:10.1186/s12967-016-0834-8) contains supplementary material, which is available to authorized users.

## Background

Type 2 diabetes (T2D) and coronary artery disease (CAD) are global public health concerns [1]. People with diabetes also have a high incidence of CAD [2]; T2D patients with CAD (T2D&CAD) have mortality rates that are about 2 to 4 times higher than those of T2D patients without CAD [3]. Recently, An et al. [4] reported that in Chinese adults, diabetes is associated with a substantially increased risk of cardiovascular-cause mortality. Thus, it is important to explore risk factors of T2D, CAD and T2D&CAD: these factors include obesity, metabolic syndrome, family history of T2D or CAD, impaired glucose tolerance, low physical activity, increased plasma triglycerides (TG), and decreased HDL-cholesterol [5–7]. Of these risk factors, plasma fatty acid composition is of particular interest because of the role of plasma fatty acids in normal and pathophysiologic responses [8].

The desaturase enzymes are of great importance in the chemical structure and functions of fatty acids. The stearoyl coenzyme is definitely required for the conversion of saturated fatty acids into monounsaturated fatty acids, and delta-5 desaturase (D5D) and delta-6 desaturase (D6D) catalyze the rate-limiting steps in the conversion of linoleic acid (LA, C18:2n-6) and gamma-linolenic acid (GLA, C18:3n-6) into long-chain n-6 and n-3 polyunsaturated fatty acids. In fact, the influence of desaturase enzymes on fatty acids has been identified as an essential factor of T2D and CAD [9]. The fatty acid desaturase genes *FADS1* and *FADS2* code, respectively, for the desaturase enzymes D5D and D6D, which cluster in a head-on-head direction on chromosome 3. The role of *FADS3* in fatty acid metabolism is still unclear, although *FADS3* is also clustered at the same location as *FADS1* and *FADS2* [10].

Previous studies have suggested that plasma and tissue concentrations of n-3 and n-6 fatty acids are associated with several single nucleotide polymorphisms (SNPs) in the *FADS1* and *FADS2* genes [11–13]. Our previous research demonstrated that participants with the rs174460 C allele had a higher risk of CAD than those who had the corresponding T allele, and that the rs174537 T allele is associated with a lower risk of CAD when compare with the carriers of rs174537 G allele [14]. Genome-wide association studies in humans have also highlighted the influence of variations in the *FADS1* and *FADS2* gene cluster on glucose and lipid metabolisms, such as total cholesterol [15] and LDL-cholesterol, and disease conditions such as metabolic syndrome, myocardial infarction, and dyslipidemia [16, 17]. However, few studies have specifically focused on the association between genetic polymorphisms in the *FADS* gene cluster and the risk of T2D&CAD. Due to the functions of *FADS* gene in fatty acids metabolism and homeostasis, we hypothesize that SNPs in *FADS* gene will influent the desaturase activity, which therefore alters the characteristics of plasma fatty acids and the risk of T2D&CAD. To test our hypothesis, in this study, we explored genetic polymorphisms in the *FADS* gene cluster and plasma fatty acids in patients with T2D, CAD or T2D&CAD, and healthy controls.

## Methods

### Patients

872 unrelated individuals were recruited at Zhongnan Hospital of Wuhan University, including: 234 patients with T2D, 200 patients with CAD, 185 patients with T2D&CAD, and 253 healthy controls. T2D was diagnosed according to 2012 ADA diabetes treatment guidelines [18]. CAD patients were with either coronary angiography or myocardial infarction, which was defined as a ≥50 % stenosis in any major coronary artery [19]. Healthy controls were randomly selected from physical examination population who had normal liver function and kidney function, and fasting blood glucose and lipids were within reference ranges; and exclusion criteria were diabetes, cardiovascular disease or other serious disease or use of any medicine or fish oil supplement. Healthy controls should maintain their usual diet, T2D patients should maintain low-fat and -carbohydrate diet, CAD patients should maintain low-fat and -sodium diet, T2D&CAD patients should maintain low-fat, -carbohydrate and -sodium diet. All patients underwent statins for cholesterol-lowering. Written informed consent was obtained from all participants, and the study protocol was approved by the ethics committee of Zhongnan Hospital of Wuhan University.

### Fatty acids analysis

To detect fatty acid methyl esters, we used a previously described method [20] with minor modifications. After filtration, 1μL of sample was injected into the Agilent 7890/5975 gas chromatography–mass spectrometry system (Agilent Technologies, Santa Clara, CA, USA). The HP-INNOWax column (30 m × 0.25 mm × 0.25 μm) (Agilent Technologies) was used for gas chromatography–mass spectrometry analysis. The gas chromatography oven temperature was programmed to increase from 60 °C (2 min) to 160 °C at 20 °C/min and then to 240 °C (6 min) at 10 °C/min, and the flow rate of carrier gas was at 1 mL/min. The interface temperature was 250 °C, the ion-source temperature was 230 °C, and the electron-impact ionization was 70 eV, with a full scan ranging from 20 to 550 m/z and a solvent delay of 3 min. Peak identification of target compounds was based on the retention times and full scan spectra of the standards. The levels of individual fatty acids are expressed as a percentage of total fatty acid methyl esters. D5D activity was estimated as the ratio of AA to DGLA, and D6D activity was estimated as the ratio of GLA to LA. Palmitoleic acid (C16:1) to palmitic acid (C16:0) and OA to SA ratios were used as a surrogate estimation of the activity of D9D-16 and D9D-18, respectively.

### SNP selection and genotyping

We used a commercially available DNA isolation kit (TIANamp, Beijing, China) to extract genomic DNA from whole blood (200 μL), according to the protocol. SNPs in FADS gene were identified using the International HapMap Project SNP database and Tag SNPs (tSNPs) were selected with their features in Haploview V4.1 by a minor allele frequency (MAF >5 %) and pairwise tagging (r^2^ ≥ 0.8), and reference population was CHB. Moreover, we examined linkage disequilibrium (LD) between any two of four selected tSNPs using the SNP Annotation and Proxy Search (SNAP) database. Four SNPs (Flap structure-specific endonuclease-10154 rs174537G>T, *FADS2* rs174616C>T, rs174460T>C, and rs174450A>C) were genotyped with the high-resolution melting of a small amplicon, as described previously [14].

### Statistical analysis

All statistical analyses were performed with SPSS 19.0 for Windows (IBM, Armonk, NY, USA). Continuous variables are expressed as means ± SDs. Skewed variables are described by the median and interquartile range. K-independent nonparametric analysis was used to compare the fatty acid levels among the T2D, CAD, T2D&CAD, and healthy groups. Ordinal logistic regression analysis (ordinal values were 1 for healthy controls, 2 for patients with T2D or CAD, and 3 for patients with T2D&CAD) were used to evaluate the associations of SNPs with diseases. Hardy–Weinberg equilibrium, genotype and allele frequency distributions were performed using SNPStats (Barcelona, Spain) [21] after adjusting for age and sex. All statistical tests were two-sided, and *p* values of less than 0.05 or Bonferroni correction-adjusted *p* values of less than 0.0125 were considered statistically significant.

## Results

### Clinical characteristics

The demographic and clinical characteristics of T2D, CAD, and T2D&CAD patients and healthy controls are listed in Table 1. We found significant differences in plasma TG, TC, HDL-cholesterol, LDL-cholesterol, and fasting plasma glucose (FPG) levels among four groups. Besides, T2D patients had the highest FPG and HbA_1c_ levels, yet CAD patients and T2D&CAD patients had lower levels of TC and LDL-cholesterol than that in T2D patients.

**Table 1:** Clinical characteristics and Differential fatty acid levels and desaturase activities in healthy controls and patients

Characteristics	Healthy controls (n = 253)	T2D patients (n = 234)	CAD patients (n = 200)	T2D&CAD patients (n = 185)	*p* ^a^
Male/Female (%)	60.5/39.5	62/38	55.5/44.5	63.2/36.8	0.412
Age (year)	59.73 ± 10.06	57.74 ± 12.76	59.47 ± 10.53	60.30 ± 9.73	0.282
Systolic blood pressure (mmHg)	128.19 ± 20.59	130.90 ± 17.73	129.16 ± 18.94	131.87 ± 19.54	0.247
Diastolic blood pressure (mmHg)	78.42 ± 13.75	76.64 ± 10.71	78.51 ± 12.20	78.61 ± 11.78	0.513
Total cholesterol (mmol/l)	4.43 (3.97, 4.91)	4.68 (3.99, 5.31)	4.05 (3.33, 4.64)	4.07 (3.31, 4.99)	<0.0001
Triglyceride (mmol/l)	1.01 (0.78, 1.32)	1.82 (1.21, 2.74)	1.26 (0.93, 1.60)	1.37 (1.01, 1.98)	<0.0001
HDL-cholesterol (mmol/l)	1.29 (1.12, 1.49)	1.05 (0.90, 1.20)	1.17 (0.99, 1.35)	1.03 (0.87, 1.29)	<0.0001
LDL-cholesterol (mmol/l)	2.70 (2.33, 3.05)	2.70 (2.10, 3.21)	2.42 (1.72, 2.89)	2.42 (1.84, 3,12)	<0.0001
Fasting plasma glucose (mmol/l)	4.92 (4.59, 5.33)	8.50 (6.45, 11.73)	5.68 (5.11, 6.29)	6.93 (6.03, 8.16)	<0.0001
HbA1c (%)	_	7.70 (6.50, 9.48)	_	6.70 (6.10, 7.30)	<0.0001
HbA1c (mmol/mol)	_	60.66 (47.54, 80.11)	_	49.73 (43.17, 56.28)	<0.0001
Fatty acids (%)					
Palmitic acid, C16:0	22.41 (21.40, 23.63)	23.61 (21.78, 25.34)	23.17 (21.38, 24.64)	23.40 (21.76, 24.77)	<0.0001
Stearic acid, C18:0	9.33 (8.52, 9.98)	9.14 (7.63, 10.78)	9.16 (8.37, 9.89)	8.96 (8.14, 9.85)	0.048
Total monounsaturated fatty acid	16.05 (14.14, 18.12)	19.27 (16.93, 21.71)	17.40 (15.47, 19.60)	18.47 (15.99, 20.65)	<0.0001
Palmitoleic acid, C16:1	0.70 (0.52, 0.95)	0.88 (0.42, 1.50)	0.95 (0.66, 1.23)	0.90 (0.59, 1.55)	<0.0001
Oleic acid, C18:1n-9	14.85 (13.10, 16.74)	18.29 (16.06, 20.52)	15.90 (14.29, 17.83)	17.23 (14.75, 19.65)	<0.0001
Total polyunsaturated n-3 fatty acid	3.65 (2.93, 4.31)	7.07 (4.42, 10.05)	3.40 (2.78, 4.00)	4.22 (3.20,7.25)	<0.0001
α-linolenic acid, C18:3n-3	0.55 (0.35, 0.76)	0.58 (0.13, 1.08)	0.55 (0.34, 0.78)	0.65 (0.34, 0.92)	0.195
Eicosapentaenoic acid, C20:5n-3	0.21 (0.00, 0.46)	0.87 (0.28, 1.75)	0.17 (0.00, 0.40)	0.18 (0.00, 1.17)	<0.0001
Docosahexaenoic acid, C22:6n-3	2.72 (2.17, 3.38)	4.78 (1.95, 7.40)	2.52 (2.00, 3.12)	3.22 (2.49, 5.01)	<0.0001
Total polyunsaturated n-6 fatty acid	46.32 (43.57, 49.04)	45.66 (42.34, 49.19)	44.37 (41.37, 47.46)	45.08 (41.69, 48.27)	<0.0001
Linoleic acid, C18:2n-6	35.96 (32.94, 39.49)	34.63 (30.50, 40.53)	33.11 (29.66, 36.64)	32.61 (29.36, 36.47)	<0.0001
γ-linolenic acid, C18:3n-6	0.22 (0.05, 0.42)	0.13 (0.00, 0.37)	0.32 (0.10, 0.57)	0.17 (0.00, 0.49)	<0.0001
Dihomo-γ-linolenic acid, C20:3n-6	1.35 (1.07, 1.63)	1.37 (0.16, 2.13)	1.54 (1.18, 2.03)	1.63 (1.03, 2.40)	<0.0001
Arachidonic acid, C20:4n-6	7.98 (6.68, 9.43)	7.59 (3.97, 10.82)	7.92 (6.28, 9.70)	9.18 (6.67, 11.27)	<0.0001
Desaturase activity					
C20:4n-6/C20:3n-6 (D5D)	6.22 (4.61, 7.67)	5.11 (3.71, 8.26)	5.23 (3.39, 7.60)	5.07 (3.47, 7.75)	0.051
C20:4n-6/C18:2n-6 (D6D)	0.22 (0.18, 0.27)	0.22 (0.10, 0.35)	0.24 (0.19, 0.31)	0.28 (0.19, 0.36)	<0.0001
C16:1/C16:0 (D9D-16)	0.03 (0.02, 0.04)	0.04 (0.02, 0.07)	0.04 (0.03, 0.05)	0.04 (0.03, 0.06)	0.002
C18:1n-9/C18:0(D9D-18)	1.62 (1.39, 1.90)	1.97 (1.58, 2.42)	1.77 (1.49, 2.06)	1.94 (1.67, 2.29)	<0.0001
n-3/n-6	0.08 (0.06, 0.10)	0.16 (0.09, 0.23)	0.08 (0.06, 0.09)	0.10 (0.07, 0.17)	<0.0001

^a^ *p* values derived from K-independent non-parametric analysis

### Plasma fatty acids and desaturase activities

**Fig. 1:**
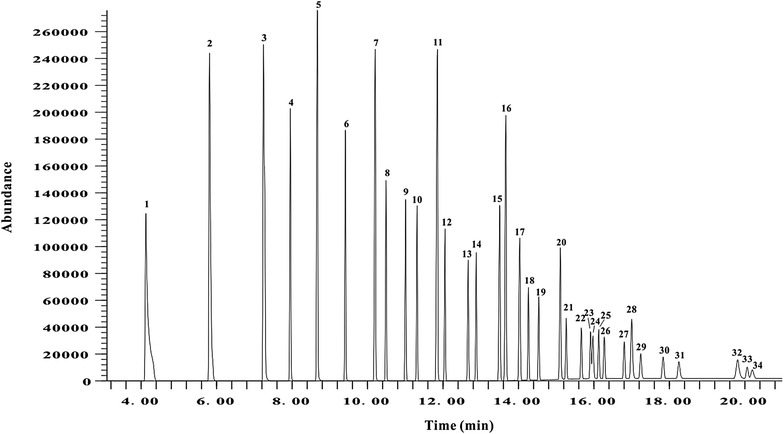
Total-ion chromatogram of standard fatty acid methyl esters separated on HP-INNOWax column. Peaks: 1, C6:0, caproic acid; 2, C8:0, caprylic acid; 3, C10:0, capric acid; 4, C11:0, undecanoic acid; 5, C12:0, lauric acid; 6, C13:0, tridecanoic acid; 7, C14:0, myristic acid; 8, C14:1, myristoleic acid; 9, C15:0, pentadecanoic acid; 10, C15:1, cis-10-pentadecenoic acid; 11, C16:0, palmitic acid; 12, C16:1, palmitoleic acid; 13, C17:0 heptadecanoic acid; 14, C17:1, cis-10-heptadecenoic acid; 15, C18:0, stearic acid; 16, C18:1n-9, oleic acid; 17, C18:2n-6, linoleic acid; 18, C18:3n-6, gamma-linolenic acid; 19, C18:3n-3, alpha-linolenic acid; 20, C20:0, arachidic acid; 21, C20:1, cis-11-eicosenoic acid; 22, C20:2, cis-11,14-eicosadienoic acid; 23, C20:3n-6, dihomo-gamma-linolenic acid; 24, C21:0, heneicosanoic acid; 25, C20:4n-6, arachidonic acid; 26, C20:3n3, cis-11,14,17-eicosatrienoic acid; 27, C20:5n-3, eicosapentaenoic acid; 28, C22:0, behenic acid; 29, C22:1n-9, erucic acid; 30, C22:2, cis-13,16-docosadienoic acid; 31, C23:0, tricosanoic acid; 32, C24:0, lignoceric acid; 33, C22:6n-3, docosahexaenoic acid; 34, C24:1, nervonic acid

As shown in Fig. 1, a total of 34 standard fatty acid methyl esters were distinctly separated on HP-INNOWax column within 20 min. Plasma fatty acids and desaturase activities, except for Alpha-linolenic acid (ALA, 18:3n-3) and D5D,differed among the four groups according to the results of K-independent nonparametric analysis (Table 1). Of the four groups, T2D&CAD patients had the highest levels of arachidonic acid (AA, C20:4n-6), dihomo-gamma-linolenic acid (DGLA, C20:3n-6) and D6D; and the lowest levels of stearic acid (SA, C18:0), LA and saturated fatty acids. Of the four groups, T2D patients had the highest levels of palmitic acid (C16:0), oleic acid (OA, C18:1n-9), EPA, DHA, monounsaturated fatty acids, n-3 fatty acids, D9D-16 (C16:1/C16:0), D9D-18 (C18:1n9/C18:0) and n-3/n-6; and the lowest levels of GLA, AA and D6D (AA/LA). Of the four groups, CAD patients had the highest levels of palmitoleic acid (C16:1) and GLA and the lowest levels of EPA, DHA, and n-3 fatty acids.

### Association of SNPs with T2D&CAD risk

Genotype distributions of the four SNPs were in Hardy–Weinberg equilibrium in healthy controls (Table 2). Among the four SNPs, only the genotype distributions of rs174537G>T differed in both additive (*p* = 0.032) and dominant models (*p* = 0.027) among all study participants. Ordinal logistic regression analysis was performed in healthy controls, T2D patients and T2D&CAD patients to assess whether rs174537 associated with the risk of T2D&CAD, the results revealed that T2D patients with rs174537 GG genotype were at higher risk of developing T2D&CAD (odds ratio (OR) 1.763; 95 % CI 1.143–2.718; *p* = 0.010) (Table 3). Besides, similar risk also existed in CAD patients with rs174537 GG genotype (OR, 2.050; 95 % CI 1.292–3.258; *p* = 0.002) (Additional file1: Table S1).

**Table 2:** Distributions of genotype and allele frequency in healthy controls and patients

SNP	Genotype	Healthy controls (n = 253)	T2D patients (n = 234)	CAD patients (n = 200)	T2D &CAD patients (n = 185)	HWE^a^	*p* value^b^	Additive model, *p* value^c^	Dominant model, *p* value^c^
rs174537G>T	GG	65	88	58	63	0.56	0.009	0.032	0.027
	GT	131	106	90	91				
	TT	57	40	52	31				
rs174616C>T	CC	113	104	88	99	0.59	0.383	0.445	0.180
	TC	115	109	90	68				
	TT	25	21	22	18				
rs174460C>T	TT	151	129	106	112	0.56	0.185	0.136	0.344
	TC	91	81	78	56				
	CC	11	24	16	17				
rs174450A>C	AA	101	86	82	80	0.46	0.550	0.743	0.589
	AC	122	113	96	80				
	CC	30	35	22	25				

^a^Hardy–Weinberg Equilibrium (HWE) was calculated in healthy controls

^b^ *p* values derived from the Chi square test of allele frequency

^c^ *p* values derived from the Chi square test of genotype distribution

**Table 3:** Risk estimation of SNPs by ordinal logistic regression analysis in healthy controls, T2D patients, T2D&CAD patients^a^

SNP	Group	Estimate	Wald value	*p* value^b^	OR (95 % CI)
rs174537G>T	GG	0.567	6.576	0.010	1.763 (1.143–2.718)
	GT	0.299	2.145	0.143	1.349 (0.904–2.014)
rs174616C>T	CC	0.256	0.932	0.334	1.292 (0.768–2.175)
	CT	−0.143	0.285	0.593	0.867 (0.513–1.464)
rs174450A>C	AA	0.054	0.052	0.820	1.055 (0.663–1.680)
	AC	0.006	0.001	0.978	1.006 (0.637–1.590)
rs174460C>T	TT	0.336	1.388	0.239	1.399 (0.800–2.447)
	TC	−0.162	1.006	0.316	0.850 (0.619–1.168)

^a^Ordinal values were 1 for healthy controls, 2 for patients with T2D, and 3 for patients with T2D&CAD

^b^ *p* values derived from ordinal logistic regression after adjustment for sex, age, TC, TG, HDL-cholesterol, LDL-cholesterol, and FPG

### Clinical characteristics, plasma fatty acids and desaturase activities in rs174537 GG genotype participants

Compared with T2D&CAD patients with rs174537 GG genotype, healthy controls with GG genotype had lower levels of TC, TG, LDL-cholesterol, FPG, C16:0, PA, OA, ALA, DHA, DGLA, AA, n-3 fatty acids, n-6 fatty acids, monounsaturated fatty acid, D6D, D9D-18, and n-3/n-6 and had higher levels of HDL-cholesterol, SA and LA. CAD patients with GG genotype had higher levels of HDL-cholesterol and lower levels of FPG, OA, DHA, n-3 fatty acids, D9D-18, and n-3/n-6 than did T2D&CAD patients with GG genotype. T2D patients with GG genotype had higher levels of TC, TG, FPG, OA, EPA, LA, n-3 fatty acids, D5D and n-3/n-6 and had lower levels of LDL-cholesterol, and D6D than did T2D&CAD patients with GG genotype (Table 4). Moreover, we also compared healthy controls, T2D, CAD with T2D&CAD patients with rs174537 GT or TT genotype (Additional file 1: Table S2, Table S3).

**Table 4:** Comparisons of clinical parameters, plasma fatty acids and desaturase activities grouping by rs174537 GG genotype

Characteristics	Healthy controls GG (n = 65)	T2D patients GG (n = 88)	CAD patients GG (n = 58)	T2D&CAD patients GG (n = 63)
Total cholesterol (mmol/l)	4.50 ± 0.72*	4.79 (4.00, 5.30)*	4.05 (3.29, 4.60)	4.52 (3.59, 5.50)
Triglyceride (mmol/l)	0.97 (0.79, 1.22)*	1.71 (1.21, 2.33)*	1.31 (1.01, 1.60)	1.36 (1.05, 1.94)
HDL-cholesterol (mmol/l)	1.37 (1.10, 1.55)*	1.07 (0.90, 1.21)	1.10 (0.94, 1.37)*	1.10 (0.93, 1.35)
LDL-cholesterol (mmol/l)	2.74 (2.27, 3.29)*	2.81(2.10, 3.17)*	2.42 (1.83, 2.84)	2.75 (2.03, 3.53)
Fasting plasma glucose (mmol/l)	4.89 (4.58, 5.54)*	8.51 (6.56, 12.24)*	5.47 (5.01, 6.29)*	7.00 (6.08, 8.24)
Total saturated fatty acid	32.14 (31.21, 33.53)	32.88 (31.24, 34.39)	33.07 (31.21, 34.97)	32.86 (29.45, 34.20)
Palmitic acid, C16:0	22.17 (21.44, 23.58)*	23.62 (21.80, 25.37)	22.46 (21.27, 24.41)	23.96 (21.00, 25.09)
Stearic acid, C18:0	9.19 (8.51, 9.87)*	9.02 (7.62, 10.55)	9.24 (8.56, 10.19)	8.45 (6.86, 9.75)
Total monounsaturated fatty acid	16.13 (14.40, 18.27)*	18.93 (16.80, 21.12)	16.58 (15.04, 19.52)	18.21 (15.41, 20.39)
Palmitoleic acid, C16:1	0.73 (0.57, 0.96)*	0.65 (0.33, 1.30)	0.88 (0.55, 1.26)	1.00 (0.55, 1.67)
Oleic acid, C18:1n-9	15.09 (13.33, 16.89)*	18.00 (16.16, 20.07)*	15.38 (13.91, 17.65)*	16.83 (13.76, 18.70)
Total polyunsaturated n-3 fatty acid	3.92 (3.24, 4.56)*	6.74 (1.99, 10.07)*	3.40 (2.81, 4.09)*	5.37 (3.45, 8.51)
Alpha-linolenic acid, C18:3n-3	0.66 (0.47, 0.86)*	0.56 (0.12, 1.13)	0.59 (0.36, 0.79)	0.67 (0.33, 0.95)
Eicosapentaenoic acid, C20:5n-3	0.31 (0.10, 0.56)	0.75 (0.21, 1.52)*	0.28 (0.00, 0.47)	0.27 (0.00, 1.80)
Docosahexaenoic acid, C22:6n-3	2.94 (2.33, 3.36)*	4.32 (1.16, 6.91)	2.50 (1.98, 3.12)*	3.66 (2.72, 5.92)
Total polyunsaturated n-6 fatty acid	45.70 (43.71, 48.00)*	46.51 (43.40, 50.40)	43.94 (41.11, 48.44)	45.85 (41.87, 48.23)
Linoleic acid, C18:2n-6	36.21 (33.33, 39.64)*	35.13 (30.12, 45.38)*	32.59 (29.13, 35.74)	32.52 (27.44, 36.37)
Gamma-linolenic acid, C18:3n-6	0.24 (0.09, 0.32)	0.12 (0.01, 0.34)	0.38 (0.11, 0.58)	0.12 (0.00, 0.54)
Dihomo-gamma-linolenic acid, C20:3n-6	1.39 (1.11, 1.77)*	1.29 (0.10, 2.12)	1.67 (1.19, 2.13)	1.64 (0.93, 2.58)
Arachidonic acid, C20:4n-6	7.66 (6.24, 9.19)*	8.04 (3.97, 11.93)	8.13 (6.33, 10.43)	9.21 (6.50, 11.48)
Desaturase activity				
C20:4n-6/C20:3n-6 (D5D)	5.54 (4.39, 7.30)	5.92 (4.33, 8.41)*	5.09 (3.47, 7.78)	4.20 (2.66, 6.85)
C20:4n-6/C18:2n-6 (D6D)	0.22 (0.16, 0.26)*	0.23 (0.09, 0.39)*	0.24 (0.19, 0.34)	0.29 (0.19, 0.38)
C16:1/C16:0 (D9D-16)	0.03 (0.02, 0.05)	0.03 (0.01, 0.06)	0.04 (0.03, 0.06)	0.04 (0.03, 0.07)
C18:1n-9/C18:0(D9D-18)^1^	1.64 (1.39, 2.00)*	1.86 (1.56, 2.43)	1.73 (1.44, 2.06)*	1.95 (1.69, 2.32)
n-3/n-6	0.08 (0.07, 0.10)*	0.15 (0.04, 0.23)*	0.08 (0.06, 0.09)*	0.11 (0.07, 0.21)

* p < 0.0125 derived from two-independent nonparametric analysis with Bonferroni correction (T2D&CAD patients vs healthy controls or T2D patients or CAD patients)

## Discussion

In this study, we found that either plasma fatty acid levels or the estimated desaturase activities significantly differed in T2D, CAD, T2D&CAD patients and healthy controls; T2D patients with rs174537 GG genotype were at risk of developing T2D&CAD. Additionally, T2D&CAD patients with the rs174537 GG genotype had elevated plasma levels of TC, LDL-cholesterol, FPG, GLA, DGLA, and AA.

Interestingly, T2D patients had the highest EPA and DHA concentrations, whereas CAD patients had the lowest levels of plasma EPA and DHA. T2D&CAD patients showed increased D6D (AA/LA), D9D-18 (OA/SA), and D9D-16 (C16:1/C16:0) activity.

Our findings, to some extent, suggest that elevated levels of EPA and DHA might protect T2D patients from CAD. Several large-scale studies of fish oil supplements that contain high concentrations of EPA and DHA have also confirmed the beneficial effects of n-3 fatty acids on cardiovascular events [22, 23]. Other studies showed that fish oil supplements reduced the number of deaths and episodes of chronic cardiac failure [24] and patients treated with EPA had a 22 % lower risk of CAD than those not treated with EPA [2], which confirmed that high blood EPA levels,compare to DHA, can reduce cardiovascular events [25].

Our data further support these conclusions that EPA can prevent coronary events. Moreover, one study found that EPA suppresses TG synthesis in the liver and lowers serum TG and decreases atherogenic lipoproteins such as remnants and small, dense LDL-cholesterol and concluded that EPA was effective in reducing the incidence of CAD events for patients with dyslipidemia [26]. However, another study found that daily n-3 fatty acid supplements (containing EPA and DHA) did not reduce the rate of death from cardiovascular causes or other outcomes in patients at high risk for cardiovascular events [27]. We also found that CAD patients with or without T2D had lower levels of TC and LDL-cholesterol than did T2D patients. Clinical studies [28, 29] have shown that lipid-lowering therapy, such as statins, plays an extremely important role in preventing and treating CAD, and statins can competitively inhibit 3-hydroxy-3-methylglutaryl-coenzyme A reductase activity and can reduce hepatic cholesterol synthesis, circulating LDL-cholesterol levels, and other apolipoprotein B-containing lipoproteins [30, 31].

We found that T2D patients had the highest D9D-16 and D9D-18 levels, followed by T2D&CAD patients. Moreover, T2D&CAD patients had the lowest D5D activity and the highest D6D activity. Increased D9D activity is associated with insulin resistance, fatty liver disease, and metabolic syndrome, and D9D is considered to be a promising target for treating insulin resistance [32]. Kröger et al. [33] discovered that the estimated D5D activity was negatively correlated with diabetes risk, whereas the estimated D6D activity was strongly and positively correlated with diabetes risk. Several studies have suggested that participants with a high estimated D5D activity have a 50 % lower risk of diabetes compared with those with a low D5D activity; these findings were based on analysis of extreme quintiles [34, 35] or tertiles [36]. CAD patients with elevated D6D activity had a higher risk of diabetes [37]. Although the mechanism by which insulin resistance increases cardiovascular risk is still unclear, D5D and D6D, which are encoded by *FADS1* and *FADS2*, may influence glucose metabolism [38]. Several studies have reported that SNPs in the *FADS* gene cluster are correlated with desaturase activity and that this correlation differed among ethnic groups [15, 39].

As we all know, cardiac and vascular diseases are often accompanied by diabetes, however, in some patients who had had a CAD but who had not previously been diagnosed with diabetes. Obviously, the severity of healthy control, T2D, and T2D&CAD is gradually increased, which is a typical ordinal event of T2D progress. Thus, we use ordinal logistic regression to find out whether T2D patients with rs174537 GG genotype were at risk of developing T2D&CAD. Our results demonstrate that the genotype distributions of rs174537G>T influence desaturase activity and are associated with the risk of both T2D and CAD, which is consistent with previous reports [11, 40]. Besides, in order to exclude the impact of CAD, we set up a CAD group, therefore the ordinal logistic regression was also applied to the healthy control, CAD and CAD&T2D and the results are listed in Additional file 1: Table S1. We also found that the minor allele T of rs174537 was significantly associated with n-3 fatty acid concentrations in the three patient groups. Numerous SNPs in the *FADS* gene cluster were reported to be significantly associated with fatty acid alterations in serum and red blood cell membranes [16, 30, 41]. Genome-wide association studies analysis also confirmed that the rs174537 SNP is significantly associated with the AA level in patients with CAD and/or T2D [16]. However, in our study, we observed this association only in the T2D&CAD group. The rs174537 SNP is located in an intron and is in linkage disequilibrium with rs174546 (r^2^ = 0.99) and rs3834458 (r^2^ = 0.98), which can influence gene expression directly [17, 42]. Therefore, it is possible that this variant of the rs174537 SNP is a marker of other functional polymorphisms or is in linkage with currently unidentified causal variants that affect fatty acid concentrations. In addition, T2D&CAD patients with the rs174357 GG genotype had decreased D5D and elevated D6D and D9D activity. Our previous study of CAD patients also supports this finding [14].

Indeed, we were unable to take “one-size-fits-all” medication therapy for patients and standard energy intake for all subjects, that resulted in failure to adjust for dietary intake and medicine in the statistical analyses, and our HP-INNOWax column assay did not get the trace amounts of several fatty acids in human plasma, but with a large sample size, these factors did have limited influence on our research. Our data indicate that polyunsaturated fatty acid metabolisms, desaturase activity, and *FADS* polymorphisms contribute to the simultaneous development of T2D and CAD. Our results suggest that rs174537 may be a key SNP in the *FADS* gene cluster and is associated with plasma fatty acid levels.

## Conclusions

It is the first time to show an association of plasma fatty acids, desaturase activities, and *FADS* genotypes with patients who had both T2D and CAD. Genetic variation in the *FADS* gene cluster, particularly rs174537, might cause the alterations in plasma fatty acids and desaturase levels, which could provide new insights for diagnostic and therapeutic strategies for patients with both T2D and CAD, and help prevent T2D patients from CAD.

## Additional file

10.1186/s12967-016-0834-8 Online Supplemental Tables.
