# Supplementary material for: Polymorphisms in FADS1 and FADS2 alter plasma fatty acids and desaturase levels in type 2 diabetic patients with coronary artery disease
Source: J Transl Med. 2016 Mar 22;14:79. doi: 10.1186/s12967-016-0834-8 (PMC4802592; doi:10.1186/s12967-016-0834-8)
Supplement: Supplementary file 1 — 10.1186/s12967-016-0834-8 Online Supplemental Tables. [file 12967_2016_834_MOESM1_ESM.doc]

**Online Supplemental Materials**

**Table S1** Ordinal logistic regression analysis of SNPs in healthy controls, CAD patients and T2D&CAD patients ^1^.

| SNP | Group | Estimate | Wald value | *p* value^2^ | OR(95% CI) |
| --- | --- | --- | --- | --- | --- |
| rs174537G>T | GG | 0.718 | 9.264 | 0.002 | 2.050(1.292 – 3.258) |
|  | GT | 0.422 | 3.812 | 0.051 | 1.525(0.998 – 2.330) |
| rs174616C>T | CC | 0.306 | 1.151 | 0.283 | 1.358(0.776 – 2.375) |
|  | CT | 0.039 | 0.019 | 0.891 | 1.040(0.591 – 1.829) |
| rs174450A>C | AA | 0.354 | 1.688 | 0.194 | 1.425(0.835 – 2.428) |
|  | AC | 0.035 | 0.017 | 0.898 | 1.036(0.610 – 1.758) |
| rs174460C>T | TT | 0.097 | 0.087 | 0.768 | 1.102(0.579 – 2.098) |
|  | TC | -0.266 | 2.309 | 0.129 | 0.766(0.543 – 1.080) |

^1^ Ordinal values were 1 for healthy controls, 2 for patients with CA and 3 for patients with T2D&CAD.

^2^ *P* values derived from ordinal logistic regression after adjustment for sex, age, TC, TG, HDL -cholesterol, LDL-cholesterol and FPG.

**Table S2 Comparisons of clinical parameters, plasma fatty acids and desaturase activities grouping by rs174537 GT genotype**

| **Characteristics** | **Healthy controls**  **GT (n=131)** | **T2D patients**  **GT (n=106)** | **CAD patients**  **GT (n=90)** | **T2D&CAD patients**  **GT (n=91)** |
| --- | --- | --- | --- | --- |
| Total cholesterol (mmol/l) | 4.46(4.05, 4.96) * | 4.67(4.00, 5.31) * | 4.05(3.42, 4.75) | 3.99(3.32, 4.73) |
| Triglyceride (mmol/l) | 1.00(0.75, 1.38) * | 1.87(1.21, 2.98) * | 1.29(0.84, 1.75) | 1.35(0.99, 2.02) |
| HDL-cholesterol (mmol/l) | 1.27(1.13, 1.50) * | 1.05(0.90, 1.19) | 1.17(1.03, 1.33) * | 1.00(0.83, 0.29) |
| LDL-cholesterol (mmol/l) | 2.73±0.53* | 2.69±0.87* | 2.41±0.87 | 2.35±0.87 |
| Fasting plasma glucose (mmol/l) | 4.92(4.59, 5.28) * | 9.61(6.41, 11.98) * | 5.72(5.11, 6.29) * | 6.55(5.64, 7.85) |
| Total saturated fatty acid | 32.83(31.44, 34.59) | 32.83(31.65, 34.98) | 33.44(31.32, 35.66) | 32.77(31.37, 34.29) |
| Palmitic acid, C16:0 | 22.45(21.40, 23.95) * | 23.55(21.61, 25.05) | 23.38(21.94, 24.88) | 23.51(22.09, 24.95) |
| Stearic acid, C18:0 | 9.37(8.52, 10.00) | 9.40(7.83, 11.54) | 9.11(8.19, 9.82) | 9.13(8.42, 10.01) |
| Total monounsaturated fatty acid | 16.10(13.90, 17.81) * | 18.92(16.65, 21.51) | 17.88(16.22, 20.90) | 19.05(16.26, 26.16) |
| Palmitoleic acid, C16:1 | 0.67(0.51, 0.930) * | 0.95(0.48, 1.62) | 1.02(0.69, 1.32) | 0.82(0.58, 1.52) |
| Oleic acid, C18:1n-9 | 14.80(12.94, 16.34) * | 17.59(15.67, 20.51) | 16.34(14.76, 19.18) | 14.54(15.39, 19.77) |
| Total polyunsaturated n-3 fatty acid | 3.43(2.76, 4.16) * | 7.39(5.38, 10.52) * | 3.34(2.78, 3.99) * | 3.69(2.98, 6.25) |
| Alpha-linolenic acid, C18:3n-3 | 0.49(0.33, 0.74) | 0.67(0.17, 1.05) | 0.50(0.32, 0.74) | 0.63(0.34, 0.85) |
| Eicosapentaenoic acid, C20:5n-3 | 0.20(0.00, 0.44) | 1.01(0.39, 1.85) * | 0.13(0.00, 0.34) | 0.15(0.00, 0.44) |
| Docosahexaenoic acid, C22:6n-3 | 2.63(2.10, 3.38) | 5.07(2.93, 8.69) * | 2.59(2.03, 3.12) * | 2.92(2.24, 4.69) |
| Total polyunsaturated n-6 fatty acid | 46.37(43.52, 49.46) * | 45.36(41.92, 48.32) | 43.70(41.19, 46.26) | 44.13(41.60, 47.84) |
| Linoleic acid, C18:2n-6 | 35.89(32.86, 39.51) * | 33.85(30.50, 38.59) | 33.22(29.69, 36.33) | 32.92(30.48, 30.63) |
| Gamma-linolenic acid, C18:3n-6 | 0.24(0.08, 0.44) | 0.17(0.00, 0.38) | 0.25(0.07, 0.50) | 0.18(0.00, 0.47) |
| Dihomo-gamma-linolenic acid, C20:3n-6 | 1.32(1.07, 1.61) | 1.53(1.00, 2.38) | 1.49(1.15, 2.03) | 1.55(1.06, 2.21) |
| Arachidonic acid, C20:4n-6 | 7.96(6.64, 9.42) | 8.43(4.96, 4.98) | 7.74(6.24, 9.62) | 8.88(6.74, 11.31) |
| Desaturase activity |  |  |  |  |
| C20:4n-6/C20:3n-6 (D5D) | 6.22(4.20, 7.62) | 4.76(3.70, 6.63) | 4.94(3.40, 7.67) | 5.62(3.68, 8.18) |
| C20:4n-6/C18:2n-6 (D6D) | 0.22(0.17, 0.26) * | 0.27(0.16, 0.36) | 0.23(0.20, 0.30) | 0.27(0.20, 0.35) |
| C16:1/C16:0 (D9D-16) | 0.03(0.02, 0.05) | 0.04(0.02, 0.08) | 0.05(0.03, 0.06) | 0.04(0.03, 0.05) |
| C18:1n-9/C18:0(D9D-18) ^1^ | 1.63±0.39* | 1.85±0.74 | 1.87±0.43 | 1.91±0.48 |
| n-3/n-6 | 0.08(0.06, 0.10) | 0.16(0.12, 0.24) * | 0.08(0.06, 0.09) * | 0.09(0.07, 0.14) |

**: p<0.0125* derived from Two-independent nonparametric analysis with Bonferroni correction (T2D&CAD patients vs healthy controls or T2D patients or CAD patients).

**Table S3 Comparisons of clinical parameters, plasma fatty acids and desaturase activities grouping by rs174537 TT genotype**

| **Characteristics** | **Healthy controls**  **TT (n=57)** | **T2D patients**  **TT (n=40)** | **CAD patients**  **TT (n=52)** | **T2D&CAD patients**  **TT (n=31)** |
| --- | --- | --- | --- | --- |
| Total cholesterol (mmol/l) | 4.27±0.68 | 4.72±1.06* | 3.97±1.01 | 3.92±1.09 |
| Triglyceride (mmol/l) | 1.07(0.82, 1.39) | 2.02(1.18, 2.93) | 1.21(0.96, 1.54) | 1.41(0.92, 2.03) |
| HDL-cholesterol (mmol/l) | 1.27(1.08, 1.42) * | 0.98(0.85, 1.16) | 1.17(1.01, 1.35) | 1.03(0.91, 1.07) |
| LDL-cholesterol (mmol/l) | 2.66(2.28, 2.90) | 2.68(1.94, 3.19) | 2.39(1.58, 2.80) | 2.09(1.81, 2.93) |
| Fasting plasma glucose (mmol/l) | 4.92(4.59, 5.32) * | 7.46(6.18, 9.91) | 5.82(5.29, 6.32) * | 7.81(6.29, 8.98) |
| Total saturated fatty acid | 32.59(31.16, 34.26) | 33.33(31.49, 34.87) * | 33.39(31.39, 35.24) * | 32.18(30.39, 33.09) |
| Palmitic acid, C16:0 | 22.75(21.39, 23.54) | 24.11(22.16, 25.81) | 22.91(21.15, 24.64) | 22.87(22.12, 24.16) |
| Stearic acid, C18:0 | 9.41(8.86, 10.09) | 8.64(7.38, 10.39) | 8.95(8.37, 9.80) | 8.89(8.36, 9.63) |
| Total monounsaturated fatty acid | 16.43±3.13 | 20.61±3.97* | 16.89±2.62 | 17.45±5.27 |
| Palmitoleic acid, C16:1 | 0.69(0.50, 0.94) * | 0.92(0.42, 1.51) | 0.87(0.68, 1.10) | 0.91(0.69, 1.56) |
| Oleic acid, C18:1n-9 | 15.34±3.02 | 19.53±3.58* | 15.39±2.41 | 16.27±5.02 |
| Total polyunsaturated n-3 fatty acid | 3.49(2.64, 4.32) * | 6.44(3.03, 7.83) | 3.55(2.77, 3.95) * | 5.13(3.60, 9.18) |
| Alpha-linolenic acid, C18:3n-3 | 0.52(0.29, 0.74) | 0.39(0.09, 1.05) | 0.57(0.35, 0.79) | 0.66(0.38, 1.01) |
| Eicosapentaenoic acid, C20:5n-3 | 0.16(0.00, 0.41) | 0.65(0.21, 2.40) | 0.20(0.00, 0.34) | 0.34(0.00, 1.54) |
| Docosahexaenoic acid, C22:6n-3 | 2.82(2.21, 3.50) | 3.20(1.50, 6.14) | 2.53(2.05, 3.16) * | 3.76(2.65, 8.26) |
| Total polyunsaturated n-6 fatty acid | 46.69±5.07 | 44.34±6.74 | 45.31±6.08 | 44.40±7.67 |
| Linoleic acid, C18:2n-6 | 36.56(32.58, 39.09) * | 34.70(31.81, 45.43) * | 34.21(29.85, 38.39) | 31.69(28.10, 34.84) |
| Gamma-linolenic acid, C18:3n-6 | 0.15(0.01, 0.42) | 0.11(0.01, 0.38) | 0.39(0.12, 0.60) | 0.24(0.00, 0.59) |
| Dihomo-gamma-linolenic acid, C20:3n-6 | 1.31(0.95, 1.51) * | 1.29(0.41, 1.77) * | 1.47(1.22, 2.00) | 1.81(1.36, 3.24) |
| Arachidonic acid, C20:4n-6 | 8.26±2.11 | 5.49±3.17* | 8.05±2.43 | 9.33±3.42 |
| Desaturase activity |  |  |  |  |
| C20:4n-6/C20:3n-6 (D5D) | 6.55(4.84, 8.43) | 4.62(3.21, 8.77) | 5.70(3.25, 7.18) | 4.49(2.86, 7.07) |
| C20:4n-6/C18:2n-6 (D6D) | 0.23(0.17, 0.30) | 0.16(0.06, 0.22) * | 0.24(0.18, 0.29) | 0.29(0.25, 0.37) |
| C16:1/C16:0 (D9D-16) | 0.03(0.02, 0.04) | 0.04(0.02, 0.07) | 0.04(0.03, 0.05) | 0.04(0.02, 0.06) |
| C18:1n-9/C18:0(D9D-18) ^1^ | 1.66±0.36* | 2.23±0.50 | 1.76±0.34 | 1.96±0.68 |
| n-3/n-6 | 0.08(0.06, 0.10) * | 0.14(0.06, 0.18) | 0.08(0.06, 0.09) * | 0.11(0.06, 0.22) |

**: p<0.0125* derived from Two-independent nonparametric analysis with Bonferroni correction (T2D&CAD patients vs healthy controls or T2D patients or CAD patients).
